# Safety of intramuscular influenza vaccine in patients receiving oral anticoagulation therapy: a single blinded multi-centre randomized controlled clinical trial

**DOI:** 10.1186/1471-2326-8-1

**Published:** 2008-05-29

**Authors:** Josep Casajuana, Begoña Iglesias, Mireia Fàbregas, Francesc Fina, Joan-Antoni Vallès, Rosa Aragonès, Mència Benítez, Edurne Zabaleta

**Affiliations:** 1 EAP Gòtic, Institut Català de la Salut, Passatge de la Pau 1, 08001 Barcelona, Spain; 2 SAP Garraf-Alt Penedès, Institut Català de la Salut, C. Manuel Marquès, 08800 Vilanova i la Geltrú, Spain; 3 Direcció Adjunta d'Afers Assistencials, Institut Català de la Salut, Av. Gran Via de les Corts Catalanes 587–589, 08007 Barcelona, Spain; 4 SAP Litoral de Barcelona, Institut Català de la Salut, C. Lope de Vega 138, 08005 Barcelona, Spain; 5 IDIAP Jordi Gol, Institut Català de la Salut, Av. Gran Via de les Corts Catalanes 587–589, 08007 Barcelona, Spain

## Abstract

**Background:**

Influenza vaccines are recommended for administration by the intramuscular route. However, many physicians use the subcutaneous route for patients receiving an oral anticoagulant because this route is thought to induce fewer hemorrhagic side effects. Our aim is to assess the safety of intramuscular administration of influenza vaccine in patients on oral anticoagulation therapy.

**Methods:**

Design: Randomised, controlled, single blinded, multi-centre clinical trial. Setting: 4 primary care practices in Barcelona, Spain. Participants: 229 patients on oral anticoagulation therapy eligible for influenza vaccine during the 2003–2004 season. Interventions: intramuscular administration of influenza vaccine in the experimental group (129 patients) compared to subcutaneous administration in the control group (100 patients). Primary outcome: change in the circumference of the arm at the site of injection at 24 hours. Secondary outcomes: appearance of local reactions and pain at 24 hours and at 10 days; change in INR (International Normalized Ratio) at 24 hours and at 10 days. Analysis was by intention to treat using the 95% confidence intervals of the proportions or mean differences.

**Results:**

Baseline variables in the two groups were similar. No major side effects or major haemorrhage during the follow-up period were reported. No significant differences were observed in the primary outcome between the two groups. The appearance of local adverse reactions was more frequent in the subcutaneous administration group (37,4% vs. 17,4%, 95% confidence interval of the difference 8,2% to 31,8%).

**Conclusion:**

This study shows that the intramuscular administration route of influenza vaccine in patients on anticoagulant therapy does not have more side effects than the subcutaneous administration route.

**Registration number:**

NCT00137579 at clinicaltrials.gov

## Background

Influenza vaccines are recommended for administration by the intramuscular route [1]. These recommendations are based on similar immunogenicity but less reactogenity if administered intramuscularly than subcutaneously or intradermally [2,3].

However, many physicians use the subcutaneous route for patients receiving an oral anticoagulant therapy because this route is thought to induce fewer hemorrhagic side effects, although no clinical trials support this recommendation. The number of people receiving oral anticoagulant therapy has increased over the past years as a result of a greater number of its indications; more people in this group are therefore likely to meet eligibility criteria for the influenza vaccination.

There is currently insufficient evidence to recommend the subcutaneous route in these patients. One before-after study [4](2) with 36 patients receiving warfarin showed no differences in the circumference of the arm compared to baseline. Only two randomised clinical trials have assessed the safety of the route of administration of influenza vaccine in patients receiving oral anticoagulant therapy [5,6]. Although both had small sample sizes (26 and 59), they seemed to support the safety of the intramuscular route.

Moreover, the US Centers for Disease Control and Prevention recommends [7] that influenza vaccine should be administered intramuscularly even in those patients receiving oral anticoagulant therapy, provided the doctor considers it appropriate and safe. After administration, compression at the point of injection should be applied for at least 2 minutes. This recommendation is based on a study carried out in 153 haemophilic patients vaccinated against hepatitis B [8].

To address this question, we studied the safety of the intramuscular route of administration for influenza vaccine compared to subcutaneous administration in patients receiving oral anticoagulation therapy.

## Methods

### Objective and design

We conducted a randomised, single blinded controlled clinical trial to assess the safety of the intramuscular administration of influenza vaccine in patients on oral anticoagulation therapy.

### Study population

We carried out the study in four primary care practices in Barcelona (Spain) between October and December 2003. Eligible patients were those older than 18 who were being treated with acenocoumarol or warfarin and were eligible for influenza vaccination. Patients with an INR higher than 4 at the time of enrolment or in the last two months, with a history of major bleeding, known hypersensitivity to any of the vaccine's components or diagnosed with dementia were excluded. We identified eligible patients through a computerized clinical registry search.

### Intervention

The control group received one dose of the Mutagrip^® ^0,5 ml of the 2003–2004 formulation of influenza vaccine through a preloaded syringe with a needle of 16 mm (5/8 inches) in length and 0,5 mm (25-gauge) in diameter subcutaneously in the deltoid region. The intervention group received the same vaccine using the same needle and syringe combination in the same place but intramuscularly. The vaccine was administered in the patient's own practice by specifically trained health care professionals.

### Ethical approval

Approved by the IDIAP Jordi Gol ethics committee of the Insitut Català de la Salut.

### Randomisation and blinding

We randomised patients to either the subcutaneous or intramuscular administration group. The randomisation of patients to each group was based on a computer generated list. After obtaining written informed consent from eligible patients and collecting baseline data the patient was assigned to either group.

Health care professionals administering the vaccine could not be blinded to patients' allocation at baseline. Blinding of the professional collecting follow-up data reduced potential bias. Since patients were not informed of the route of administration, we considered them blinded to the intervention as well.

### Follow up and outcome assessment

We carried out three follow up visits: the first at the moment of vaccination, the second at 24 hours and the third at 10 days. The visits took place in the patients' own practices and were carried out by health care professionals from their practice. When a patient failed to attend a visit, three attempts were made to contact him/her by telephone at different times. If they declined a request to come to the missed visit, they were asked for the reason and if they had had any side effects.

The outcome variables were: a) presence of any major side effect. b) Change in the circumference of the arm at the point of injection to detect haemorrhage or haematoma. A measure specifically designed for the study was used to locate the point of the circumferential reading, which corresponded to the point of injection on the deltoid area. One end of the measure, which would run parallel to the longitudinal axis of the arm, was to be placed at the upper-external border of the epicondyle with the elbow flexed to 90°; the other end of the measure would show the circumference reading point. An increase of the circumference of the arm of one or more centimetres at 24 hours after the administration of the vaccine was considered clinically significant; c) presence of skin lesions at the point of injection at 24 hours and after 10 days; d) the existence of pain during the first 24 hours and after 10 days, measured with a visual analog pain scale (from zero to ten); e) the capillary blood INR was measured with Coagucheck^® ^at 24 hours and at 10 days to assess the interaction between the anticoagulant treatment and the vaccination.

### Sample size and data analysis

The sample size calculation was based on hypothesis testing to establish the difference between the subcutaneous and intramuscular administration groups in the proportion of patients with an increase of the circumference of the arm equal or higher than one centimetre. From a previous study [6] we established that the expected event rate in the subcutaneous administration group was 40%. The intramuscular administration group should not differ from this 40% by more than 50% (20%). An α = 0.05 and β = 0.20 were used. This resulted in a sample size requirement of 97 patients per group. All patients meeting the inclusion criteria from the four practices were invited to participate in the study.

Analysis was by intention to treat since each patient was analysed in his allocation group. However, only patients who completed all follow-up visits were included in the analysis, because we could not compute the outcome variables with the rest. We carried out a descriptive analysis of the baseline and follow-up data. For the categorical outcome variables, the 95% confidence intervals of the proportions difference between the two groups of the study through the statistic z was computed after checking the application conditions. We computed, for the continuous outcome variables, the 95% confidence interval of the means difference between the two groups through a t-test after checking the application conditions. In both cases, we rejected the null hypothesis of no difference when the confidence interval included the 0 value. The relative risk and number needed to harm were used as association and impact measures.

Subsequently, we made a sub-analysis to compare patients with a baseline INR lower than 2,5 against those equal or higher than 2,5 only for the intramuscular group. The same statistical methods were used as those in the main analysis.

All calculations were made with the statistical system 'R' [9].

## Results

**Figure 1:**
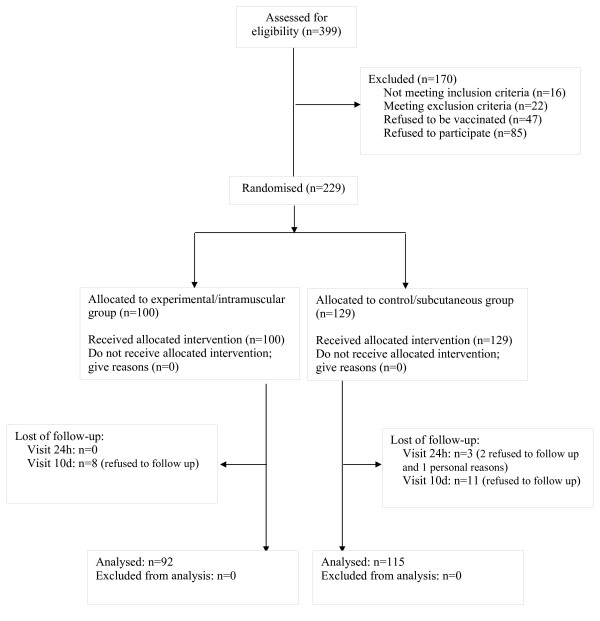
Flow chart

Three hundred ninety-nine patients were assessed for eligibility. One hundred seventy of them were excluded (Figure 1) for the following reasons: not meeting inclusion criteria (15 had been already vaccinated; 1 was not on anticoagulation therapy); meeting exclusion criteria (16 had an INR > 4, 4 had a diagnose of dementia, 1 had had a previous major bleeding, 1 was allergic to a vaccine component); refused to be vaccinated (47); or refused to participate in the study (85).

Two hundred twenty-nine patients were randomised and participated in the study (129 in the subcutaneous group and 100 in the intramuscular group). Participation rates in the practices were 70/89, 56/112, 43/102 and 60/96. All patients received the intervention to which they had been allocated.

No clinically important differences in the baseline variables were observed between the groups at the time of enrolment. Data collected from patients who refused to participate in the study did not suggest to be a subgroup with special characteristics (Table 1).

**Table 1:** Baseline data

	***Subcutaneous group (n = 129)***	***Intramuscular group (n = 100)***	***Refuse to participate (n = 132)***	***Lost of follow-up (n = 22)***
*Mean age (SD), years*	73,5 (9,7)	73,7 (9,2)	73,5 (11,0)	66,2 (17,1)

*Men, n (%)*	81 (62,8%)	56 (56,0%)	78 (59,1%)	12 (54,5%)

*Therapeutical objective, n (%)*				
*INR 2,0 – 3,0*	116 (89,9%)	94 (94,0)	128 (97,0%)	21 (95,5%)
*INR 2,5 – 3,5*	13 (10,1%)	6 (6,0%)	4 (3,0%)	1 (4,5%)

*Mean INR (SD)*	2,25 (0,59)	2,37 (0,56)		2,40 (0,61)

*INR, n (%)*				
*INR < 2*	42 (32,6%)	23 (23,0%)		5 (22,7%)
*INR >= 2 & < 3*	73 (56,6%)	63 (63,0%)		13 (59,1%)
*INR >= 3 & <= 4*	14 (10,9%)	14 (14,0%)		4 (18,2%)

*Mean arm circumference (SD), cm*	32,1 (3,5)	31,8 (4,1)		32,5 (4,2)

Most patients were taking acenocoumarol (225/229), and the rest were taking warfarin (4/229). The main diagnoses were atrial fibrillation (160/229), valvular heart disease (39/229) and ischemic heart disease (28/229). 19 patients receiving anticoagulant treatment had a metal prosthetic heart valve, so they had a higher therapeutic INR goal (2,5–3,5) as compared to the rest (2–3).

Three patients failed to attend the 24 hour visit and a further 19 the 10th day visit. Of the patients lost to follow up, 14 belonged to the subcutaneous administration group and 8 to the intramuscular group. All of them were contacted by telephone but refused to come to the follow-up visits. Follow-up variables were analyzed in 207 patients, 115 in the subcutaneous administration group and 92 in the intramuscular administration group. The baseline data of patients lost during the follow up, suggests it is a younger subgroup (Table 1).

None of the patients reported major side effects in any of the two follow-up visits, included those who failed the follow-up visits.

The circumference of the arm after 24 hours tended to increase in both groups (Table 2). Around 20% of patients in both groups had an increase of the circumference of the arm higher than one centimetre. No statistically significant differences between the groups were observed.

**Table 2:** Follow-up data

	***Subcutaneous group (n = 115)***	***Intramuscular group (n = 92)***	***95% confidence interval of the difference***
*Mean circumference of the arm (SD), cm, 24 h*	32,1 (3,4)	32,2 (4,2)	
*Mean change circumference of the arm (SD), cm, 24 h*	+0,20 (1,44)	+0,28 (0,91)	-0,40 to +0,25 *
*Patients with increase of the circumference of the arm >= 1 cm, n (%), 24 h*	27 (23,5%)	18 (19,6%)	-7,3% to +15,1%
*Patients with skin lesions in the administration area, n (%), 24 h*	43 (37,4%)	16 (17,4%)	+8,2% to +31,8%
*Erythema, n*	36	8	
*Papule, n*	0	3	
*Plaque, n*	7	5	
*Nodule, n*	0	2	
*Cyst, n*	0	0	
*Vesicle, n*	0	0	
*Blister, n*	0	0	
*Ulcer, n*	0	0	
*Scab, n*	0	2	
*Haematoma, n*	2	1	
*Pruritus, n*	4	2	
*Insensitivity, n*	1	0	
*Mean Visual Analog Pain Scale (SD), 24 h*	0,43 (1,08)	0,29 (0,84)	-0,12 to 0,41 *
*Patients with Visual Analog Pain Scale > 0, n (%), 24 h*	23 (20,0%)	13 (14,1%)	-4,3% to +16,1%
*Mean INR (SD), 24 h*	2,28 (0,64)	2,30 (0,70)	
*Mean change INR (SD), 24 h*	+0,04 (0,50)	-0,06 (0,55)	-0,04 to +0,25 *
*Mean INR (SD), 10d*	2,31 (0,67)	2,44 (0,95)	
*Mean change INR (SD), 10d*	+0,08 (0,79)	+0,07 (0,93)	-0,24 to +0,24 *

*Calculated under the checked assumption of variance homogeneity of both samples.

No patients had skin lesions in the area of injection before the vaccination. The appearance of skin lesions after 24 hours of the administration of the vaccine (Table 2) was observed in 43/115 (37,4%) patients in the subcutaneous administration group and 16/92 (17,4%) patients in the intramuscular administration group. This difference was statistically significant (95% confidence interval of the difference: 8,2% to 31,8%), representing a relative risk of 2,14 (Confidence Interval of 95% 1,30 – 3,56) and a Number Needed to Harm of 5,0 (Confidence Interval of 95% 3,1 to 11,3). The most frequent lesion observed was erythema, which appeared in 36/115 (31,3%) of the patients of the subcutaneous administration group and in 8/92 (8,7%) in the intramuscular administration group. This was followed by plaque and pruritus. Three patients presented haematoma (2 in the subcutaneous administration group and 1 in the intramuscular administration group); all of them with a basal INR between 2 and 3. All local reactions had disappeared by the 10th day after the vaccination.

Patients in the subcutaneous administration group presented higher scores in the pain scale 24 hours after vaccination (Table 2), although the mean difference was not statistically significant. A higher percentage of patients in this group marked a value other than zero in the scale, although the scores did not differ significantly to those from the intramuscular administration group. No patient reported pain at the area of the injection by the 10^th ^day.

The capillary blood INR (Table 2) tended to increase after 24 hours in the subcutaneous administration group and to decrease in the intramuscular administration group, although the difference did not reach statistical significance. This differential behaviour did not exist the 10th day after the vaccination, since in both groups an increase of the baseline INR was seen.

No differences in any of the outcome variables were found between patients with baseline INR lower and higher than 2.5 within the intramuscular administration group (Table 3).

**Table 3:** Patients in the intramuscular group by their basal INR

	***Basal INR < 2,5 (n = 55)***	***Basal INR ≥ 2,5 (n = 37)***	***95% confidence interval of the difference***
*Mean change circumference of the arm (SD), cm, 24 h*	+0,32 (1,01)	+0,22 (0,75)	-0,26 to +0,47 *
*Patients with increase of the circumference of the arm >= 1 cm, n (%), 24 h*	11 (20,0%)	7 (18,9%)	-15,4% to +17,5%
*Patients with skin lesions in the administration area, n (%), 24 h*	9 (16,4%)	7 (18,9%)	-18,5% to +13,4%
*Mean Visual Analog Pain Scale (SD), 24 h*	0,30 (0,93)	0,28 (0,70)	-0,32 to +0,36 *
*Patients with Visual Analog Pain Scale > 0, n (%), 24 h*	7 (12,7%)	6 (16,2%)	-18,3% to +11,3%
*Mean change INR (SD), 24 h*	-0,02 (0,44)	-0,13 (0,68)	-0,14 to +0,37 *
*Mean change INR (SD), 10d*	+0,14 (0,87)	-0,03 (1,01)	-0,23 to +0,58 *

*Calculated under the checked assumption of variance homogeneity of both samples.

## Discussion

Our study demonstrates the absence of clinically significant haemorrhaging following intramuscular administration of influenza vaccine in patients receiving anticoagulation therapy. Furthermore, the appearance of local lesions was significantly more frequent in patients vaccinated subcutaneously. These data appear to confirm the safety of the intramuscular route with less secondary effects than the subcutaneous route.

As a consequence of an increased number of indications for anticoagulant therapy, the number of patients receiving this treatment is increasing. This has moved patient management away from the exclusivity of certain specific services to primary care practices which makes it a first step towards a more effective and efficient management achieved by the proper patient. At the same time, the fears about possible risks, responsible for initial recommendations to avoid the intramuscular route, have been slowly overcome, and a more realistic vision has emerged. In the case of influenza vaccination, the use of a short needle and small diameter minimizes the tissue damage due to an intramuscular injection and it is fair to presume that it should not infer a substantial risk of haemorrhage.

To date, this is the largest study of the safety of the intramuscular route of the influenza vaccine in patients on anticoagulation therapy. A potential weakness is that we do not know the sample needed to detect a major secondary effect with the intramuscular route. Nevertheless this is also unknown for the subcutaneous route. One thing to consider in further investigations is to include the comorbidity and other treatments in the analysis. Another weakness is that twenty two patients were lost to follow-up, although all were contacted by telephone and none reported any event attributable to the intervention.

We can not generalize the results to other vaccines or to intramuscular injections with needles of a greater calibre. Of the most commonly used adult vaccines, tetanus toxoid and hepatitis B vaccine are marketed with needles of 25 mm long (1 inch) and 0,6 mm of diameter (23-gauge). Pneumococcal vaccine has the same diameter as influenza vaccine (0,5 mm or 25-gauge) but a greater length (25 mm). As noted in the introduction, safety data are only available for hepatitis B vaccine, although this is based on a study of haemophilic patients without a control group.

Although the sample size is small, the influenza vaccine could be administered with safety to patients with artificial valves and higher INR levels (2,5 to 3,5), as we observed the same results in the group of patients who at the time of the vaccination had an INR between 2,5 and 4 and those who had INR lower than 2,5 (Table 3).

Our results suggest that administering influenza vaccine intramuscularly in patients receiving anticoagulant therapy can be done safely, and we would support changes in recommendations to allow for intramuscular administration without compression at the site of injection. While addressing similar issues regarding other vaccines, especially the tetanus vaccine, is still necessary, we believe that intramuscular administration of influenza and hepatitis B vaccines in this population is safe.

## Competing interests

The authors declare that they have no competing interests.

## Authors' contributions

JC, BI and J–AV conceived the study and participated in its design. JC, RA and MB conducted and coordinated the trial. FF and EZ performed the statistical analysis. JC, MF and FF wrote the manuscript. All authors read and approved the final manuscript.

## Pre-publication history

The pre-publication history for this paper can be accessed here:


